# Preoperative Status of Gut Microbiota Predicts Postoperative Delirium in Patients With Gastric Cancer

**DOI:** 10.3389/fpsyt.2022.852269

**Published:** 2022-03-03

**Authors:** Hu Liu, Gao Cheng, Yuan-ling Xu, Qi Fang, Lei Ye, Chun-hui Wang, Xue-sheng Liu

**Affiliations:** ^1^Department of Anesthesiology, Key Laboratory of Anesthesiology and Perioperative Medicine of Anhui Higher Education Institutes, First Affiliated Hospital of Anhui Medical University, Anhui Medical University, Hefei, China; ^2^Department of Anesthesiology, Fourth Affiliated Hospital of Anhui Medical University, Hefei, China; ^3^Department of Neurosurgery, First Affiliated Hospital of Anhui Medical University, Hefei, China

**Keywords:** surgery, aging, gut microbiota, post-operative delirium, prediction, *Shigella*

## Abstract

**Introduction:**

Post-operative delirium (POD) is a serious complication which occurs after surgery, especially in the elderly undergoing abdominal surgery. Increasing evidence has revealed an association between the gut microbiota and psychological disorders involving the “brain-gut” axis. However, the association between the pathogenesis of POD after abdominal surgery in aging and composition of the gut microbiota remains unclear.

**Methods:**

Forty patients (≥65 years old) who underwent abdominal surgery were included in the study. Twenty patients had POD, whereas 20 patients did not. POD was diagnosed and assessed using the confusion assessment method (CAM) during the postoperative period. Total DNA fractions were extracted from all fecal samples of patients. 16S rRNA sequencing was performed to determine the composition of the gut microbiota. The quality of the samples was determined by calculating the α- and β-diversities.

**Results:**

The α- and β-diversities indicated that the samples were eligible for detection and comparison. We observed multiple differentially abundant bacteria in patients with and without POD. Generally, *Proteobacteria, Enterbacteriaceae, Escherichia shigella, Klebsiella, Ruminococcus, Roseburia, Blautia, Holdemanella, Anaerostipes, Burkholderiaceae, Peptococcus, Lactobacillus*, and *Dorea* were abundant in the POD cohort, whereas *Streptococcus equinus* and *Blautia hominis* were abundant in the control cohort. The results of receiver operating characteristic (ROC) curve analysis showed that the area under the curve (AUC) of *Escherichia shigella* was 0.75. Phenotype prediction showed that the gut microbiota may influence POD by altering the tolerance to oxidative stress.

**Conclusion:**

There were significant associations between the pathogenesis of POD and composition of the gut microbiota. *Escherichia shigella* are promising diagnostic bacterial species for predicting POD onset after abdominal surgery in elderly people.

**Clinical Trial Registration:**

http://www.chictr.org.cn/index.aspx, Chinese Clinical Trial Registry ChiCTR200030131.

## Introduction

Post-operative delirium (POD) is a neurobehavioral symptom characterized by changes in consciousness and unfocused attention (1). POD tends to occur in elderly patients with longer hospital stays and leads to a higher lethality and a lower quality of life (2, 3). As a common complication after surgery, the incidence of POD varies among surgical types and age (4, 5). POD shows a lower incidence among patients undergoing out-patient surgery (6) but a relatively higher incidence in those undergoing abdominal surgery and in elderly people (7–9). Considering the high incidence (10) and negative effects of POD, studies are needed to identify effective predictive markers and mechanisms for surgical patients in clinical practice.

The gut bacteria of adults are composed of around 10^12^–10^14^ microbes, of which the number is much higher than that of microbes on the skin and cells in the body (11). The gut microbiota participates in numerous signal transduction, metabolic pathways and the regulation of immune-inflammatory axis in the host (12, 13). Thus, it has been considered as a functional organ or second human genome in recent years (14). The gut bacteria is a promising target for investigating markers or the underlying pathogenesis of diseases. Ding et al. (15) found that gastrointestinal symptoms in children were significantly associated with the symptoms of autism spectrum disorder (ASD), and the bacteria *Actinobacteria* and *Firmicutes* may play a role in ASD pathogenesis. Recently, increasing evidence has indicated an association between the gut microbiota and neuropsychiatric diseases, termed as the “brain-gut” axis (16, 17). Although POD was shown to be significantly associated with age and inflammatory status of patients (18–20), the mechanisms of POD are still unclear.

In a previous study of POD, Zhang et al. (21) detected an association between an abnormal composition of the gut microbiota and delirium-like behaviors after abdominal surgery in mice. Additionally, Maekawa et al. (22) observed a clinical association between pseudopsia and the gut microbiota among patients who underwent cardiac surgery. However, the relationship between POD and the gut microbiota in various surgery types and the relationship with aging has not been widely examined.

Abdominal surgery dramatically alters the composition of the gut microbiota and leads to POD, at a higher incidence rate compared to other types of surgery. However, the alterations of gut bacteria after surgery may be influenced by the applications of antibiotics (23, 24), which would confound the onset of POD. We hypothesized that the preoperative gut microbiota creates a certain gut or even systemic microenvironment, and surgery-induced alterations to the gut microbiota may lead to pathological changes in the “brain-gut” axis, subsequently leading to POD.

In this study, we investigated the association between the gut microbiota composition and POD in elderly patients who underwent abdominal surgeries to identify predictive markers of POD.

## Materials and Methods

### Patients

This observational study was conducted from March 2020 to December 2020. Patients, aged 65 years and older and scheduled to undergo radical surgery for gastric cancer, with an American Society of Anesthesiologists (ASA) score of I–III and anticipated surgery time of 1.5–6 h, were included. All patients were screened with a mini-mental state examination (MMSE), and patients with scores of <20 were not enrolled because of dementia concerns. Patients were excluded if they met any of the following criteria: history of severe mental illness or dementia; extant factors that may affect cognition assessment, such as language, visual and auditory dysfunction, an unstable mental status or mental illness; and known or suspected abuse of an analgesic drug. Patients with severe adverse events during the operation (such as bleeding, anaphylactic shock, etc.) that led to death, life-threatening, irreversible damage to organ function, or prolonged hospital stay were also excluded. This study was approved by the Ethics Committee of First Affiliated Hospital of Anhui Medical University (Ethical Committee No. PJ2019-15-18), and registered in the Chinese Clinical Trial Registry (registration number ChiCTR200030131). Informed consent was obtained from all individuals, and all procedures conformed to the standards in the Declaration of Helsinki.

### Anesthetic Management

All participants underwent radical gastrectomy for gastric cancer. All surgeries were performed under general anesthesia with midazolam, sufentanil, etomidate, and cisatracurium. Anesthesia was maintained by inhalation anesthetics or intravenous anesthesia. Bispectral index values of 40–60 were consistent with general anesthesia. A titration of 0.1–0.2 μg/kg sufentanil was given intravenously before the end of surgery, and intravenous controlled analgesia was used for postoperative analgesia. Medications were used to prevent postoperative nausea and vomiting.

### Delirium Assessment

Assessment of delirium with confusion assessment method (CAM) was performed preoperatively (baseline) twice daily at 8 a.m. and 2 p.m. after the surgery until day 7. Patients were rendered as either CAM-positive (delirium present) or CAM-negative (delirium absent). The diagnosis of delirium was confirmed by psychiatrist consultation and consisted of four clinical criteria: (1) acute change and fluctuating course, (2) inattention, (3) disorganized thinking, and (4) altered level of consciousness. A diagnosis of delirium requires the presence of features 1 and 2 and either 3 or 4. Patients with hyperactive delirium were intravenously administered haloperidol in increments of 1–5 mg every 4 h as first-line treatment, which was repeated every 60 min as necessary.

### Fecal Sample Collection and Preparation

Fecal samples were collected from all patients before surgery using sterile swabs and stored at −80°C. None of the patients had been administered antibiotics within the past 6 months before sampling. Bacterial DNA was extracted using an E.Z.N.A. ®Stool DNA Kit (D4015, Omega, Inc., Norcross, GA, USA) according to the manufacturer's protocol. The total DNA was eluted in 50 μl of elution buffer and stored at −80°C until measurement using PCR by LC-BioTechnology Co., Ltd. (Hang Zhou, Zhejiang Province, China).

### PCR Amplification and 16S rDNA Sequencing

The V3-V4 region of the bacterial 16S rRNA gene was amplified with primers 341F (5′-CCTACGGGNGGCWGCAG-3′) and 805R (5′-GACTACHVGGGTATCTAATCC-3′). PCR was performed according to a previously published study (25). The amplicon pools were prepared for sequencing, and the size and quantity of the amplicon library were assessed on an Agilent 2100 Bioanalyzer (Agilent Technologies, Santa Clara, CA, USA) and using a Library Quantification Kit for Illumina (Kapa Biosciences, Woburn, MA, USA), respectively. The libraries were sequenced on NovaSeq PE250 platform (Illumina, San Diego, CA, USA).

### Data Analysis

Demographic information and intraoperative data were analyzed using SPSS 23.0 software (version 23; SPSS, Inc., Chicago, IL, USA). The normal distribution of the data was evaluated using one-sample Kolmogorov–Smirnov test. Normally distributed continuous variables were presented as the means ± standard deviations and analyzed using independent sample *t*-tests. Categorical variables were presented as numbers (frequencies) and analyzed using Pearson's chi-square or Fisher's exact tests. *P*-values were two-sided, and *P* < 0.05 was considered to indicate significant results.

An Illumina NovaSeq platform was used for sample sequencing according to the manufacturer's recommendations, provided by LC-Bio. Paired-end reads were assigned to samples based on their unique barcode and truncated by cutting off the barcode and primer sequence. Paired-end reads were merged by using FLASH. Quality filtering of the raw reads was conducted under specific filtering conditions to obtain high-quality clean tags according to fqtrim (v0.94). Chimeric sequences were filtered using Vsearch software (v2.3.4). After dereplication using DADA2, we obtained feature table and feature sequences. The α- and β-diversity were calculated using QIIME2, with the same number of sequences extracted randomly by reducing the number of sequences to the minimum of some samples, and relative abundance (X bacteria count/total count) was used in bacteria taxonomy analysis. Images were drawn by R software (v3.5.2; The R Project for Statistical Computing, Vienna, Austria). Sequence alignment for species annotation was performed using BLAST with the SILVA and NT-16S alignment databases.

## Results

### Baseline and Intra-Operative Data

The baseline demographics characteristics of the 40 patients in the POD and control group were similar in terms of age, sex, and body mass index (Table 1). Similarly, there were no significant differences in intra-operative data such as the operation time or ASA fitness grade (*P* > 0.05) (Table 1). The surgical types for the patients included laparotomy operation and endoscopic surgery, which were decided to be performed by the primary diseases and the physical condition of the patients. We reviewed some studies and found that there was no difference in the incidence of POD between the two surgical types (26, 27). Therefore, the surgical types could not be regarded as a confounding factor in our study.

**Table 1:** Patient characteristics.

	**Control group**	**POD group**	***P*-value**
Age (years)	70.90 ± 3.48	71.35 ± 4.08	0.710
Sex (M/F)	12/8	11/9	0.749
BMI (kg/m^2^)	21.25 ± 2.86	21.41 ± 2.73	0.858
ASA physical status			0.519
I	0 (0%)	0 (0%)	
II	11 (55%)	13 (65%)	
III	9 (45%)	7 (35%)	
Duration of surgery, min	171.5 ± 56.85	188.6 ± 73.39	0.414

*Continuous variables are presented as the mean ± SD; categorical variables are presented as numbers. ASA, American Society of Anesthesiologists; BMI, body mass index; F, female; M, male; SD, standard deviation*.

### Abundance of Gut Microbiota Between Patients With and Without POD

**Figure 1:**
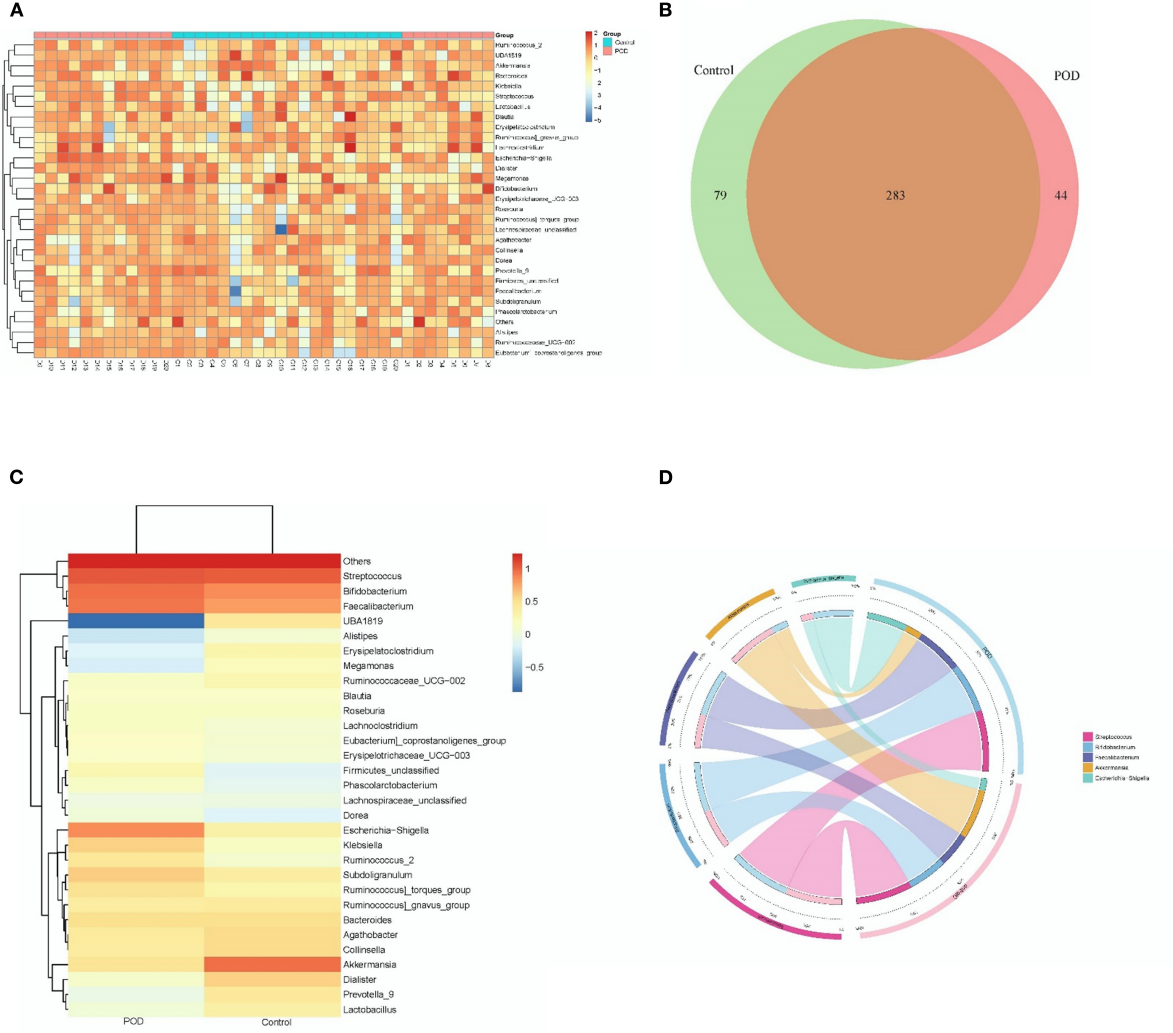
Bacterial composition analysis in different samples **(A)** and cohorts **(B)**. Venn plot depicting common and specific microbes in different cohorts **(C)**. Circos plot showing the corresponding relation between cohorts and bacterial species**(D)**.

The original contributions presented in the study are publicly available. This data can be found at: http://www.ncbi.nlm.nih.gov/bioproject/797529. We illustrated the top 30 abundant gut bacteria with using taxonomy graphics according to the patient samples (Figure 1A) and groups (Figure 1B) at the genus level. There were 283 common bacterial categories at the genus level between the POD and control cohorts. However, 44 and 79 categories of bacteria were specific in the POD and control cohorts, respectively (Figure 1C). Furthermore, we depicted correlations between the 5 top abundant microbiota (*Streptococcus, Bifidobacterium, Faecalibacterium, Akkermansia*, and *Escherichia shigella*) and the cohort in a Circos graph (Figure 1D).

### Diversity Analysis

**Figure 2:**
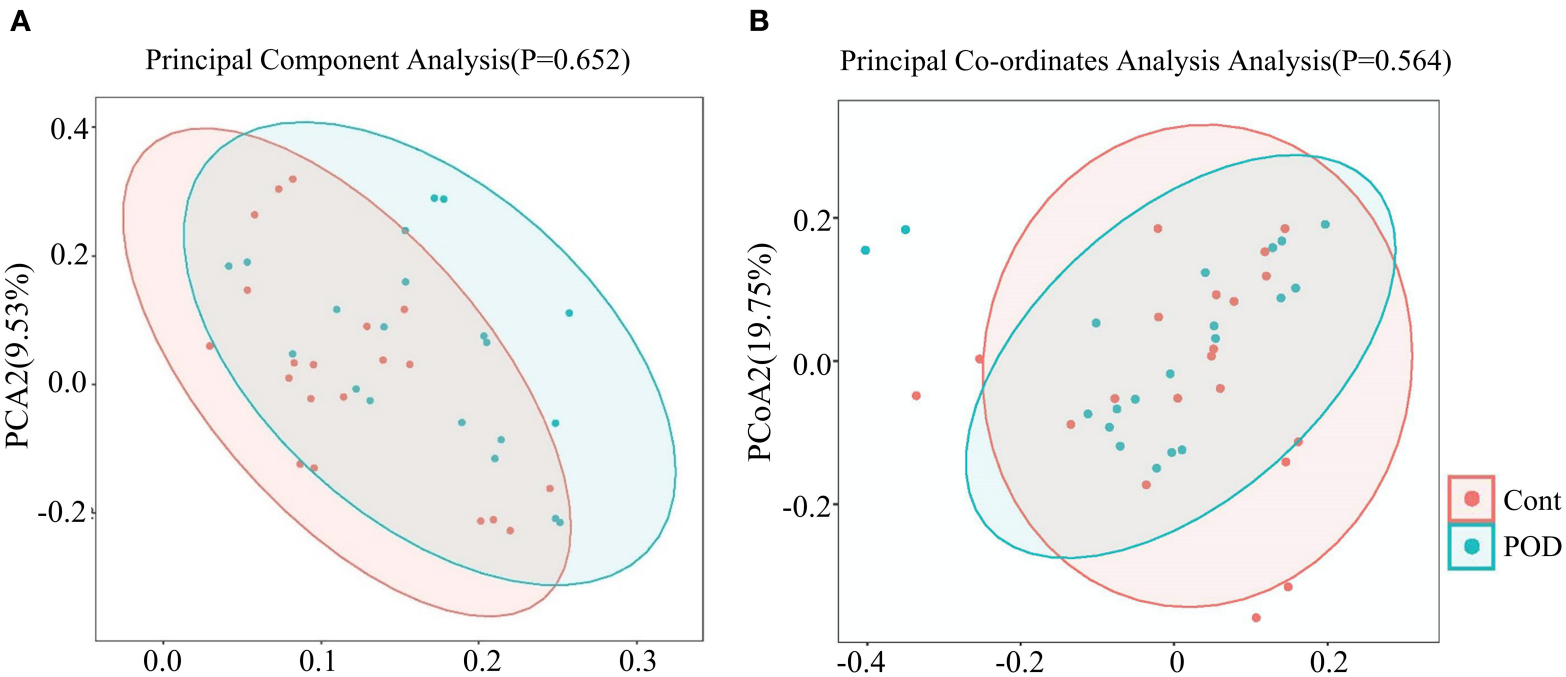
β-diversity analyses of data. **(A)** Principal component analysis (PCA). **(B)** Principal Co-ordinates Analysis (PCoA).

We analyzed the diversity for quality control of the samples in detecting the gut microbiota. Supplementary Figure 1A shows the observed operational taxonomic units between the POD and control cohorts, which did not significantly differ (*P* = 0.31). Good's coverage index indicated an excellent sequencing depth (Supplementary Figure 1B). We also calculated the Chao, Shannon, and Simpson indices to evaluate the abundance and homogeneity of the samples (Supplementary Figures 1C,E). The results indicated good sequencing quality. Principal Component Analysis (PCA) and Principal Co-ordinates Analysis (PCoA) were performed to determine the characteristics of the samples (Figures 2A,B). The results indicated that samples from two groups had comparable characteristics.

### Differential Abundance of Gut Microbiota and Diagnostic Efficacy in POD

**Figure 3:**
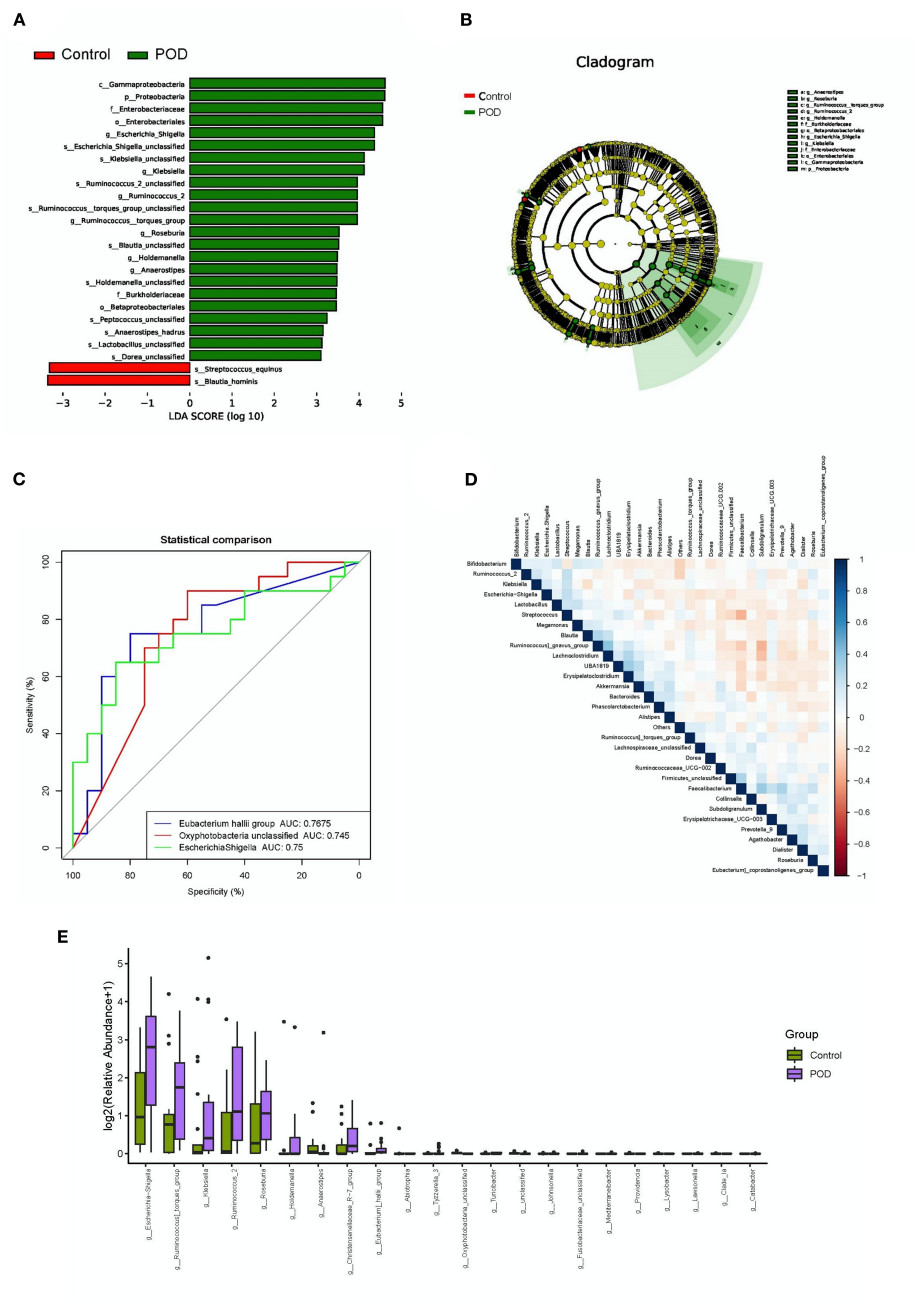
Differential abundance and diagnostic efficacy of gut bacterial between POD and control cohorts. **(A)** Linear discriminant analysis effect size (LEfSe) of differentially abundant bacteria; **(B)** Cladogram of differentially abundant bacteria; **(C)** Receiver operating characteristic curve analysis of the diagnostic efficacies of bacteria (AUC > 0.7); **(D)** Correlation analysis among bacteria; **(E)** Differentially abundant bacteria in Genus level.

We investigated the differential abundance of the gut microbiota to identify specific bacterial types that may be involved in the pathogenesis and useful for the diagnosis of POD. Analysis using linear discriminant analysis effect size (LEfSe) indicated that multiple bacterial types differed in abundance between the POD and control cohorts, with a characteristics of linear discriminant value >3 (Figures 3A,B). *Proteobacteria, Enterbacteriaceae, E. shigella, Klebsiella, Ruminococcus, Roseburia, Blautia, Holdemanella, Anaerostipes, Burkholderiaceae, Peptococcus, Lactobacillus*, and *Dorea* were more abundant in the POD cohort, whereas *Streptococcus equinus* and *Blautia hominis* were more abundant in the control cohort. Meanwhile, the difference of abundance of gut bacteria in Genus level between the POD and control cohorts was shown in Figure 3E. Moreover, a receiver operating characteristic (ROC) curve was drawn to identify bacteria useful for diagnosing POD. The area under the curve (AUC) of *Eubacterium hallii* (0.7675), *Oxyphotobacteria* (0.745), and *E. shigella* (0.75) showed areas of higher than 0.7 (Figure 3C). Based on these results, *E. shigella* was a promising predictive bacteria for diagnosing POD. No obvious correlations were observed among the different bacterial types (Figure 3D).

### Functional and Phenotype Predictions

**Figure 4:**
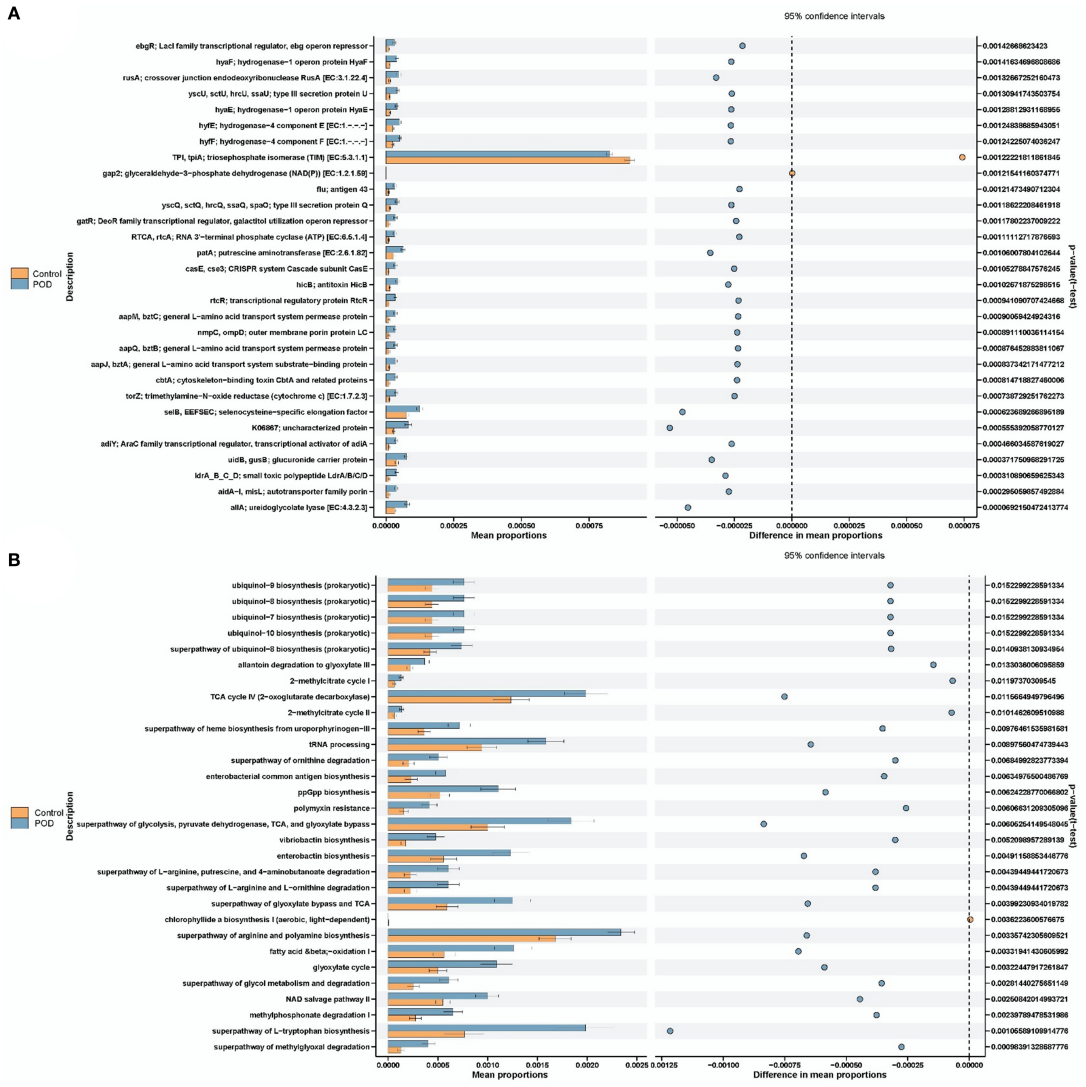
Functional prediction by phylogenetic investigation of communities by reconstruction of unobserved states. **(A)** KO prediction. **(B)** Pathway abundance prediction.

**Figure 5:**
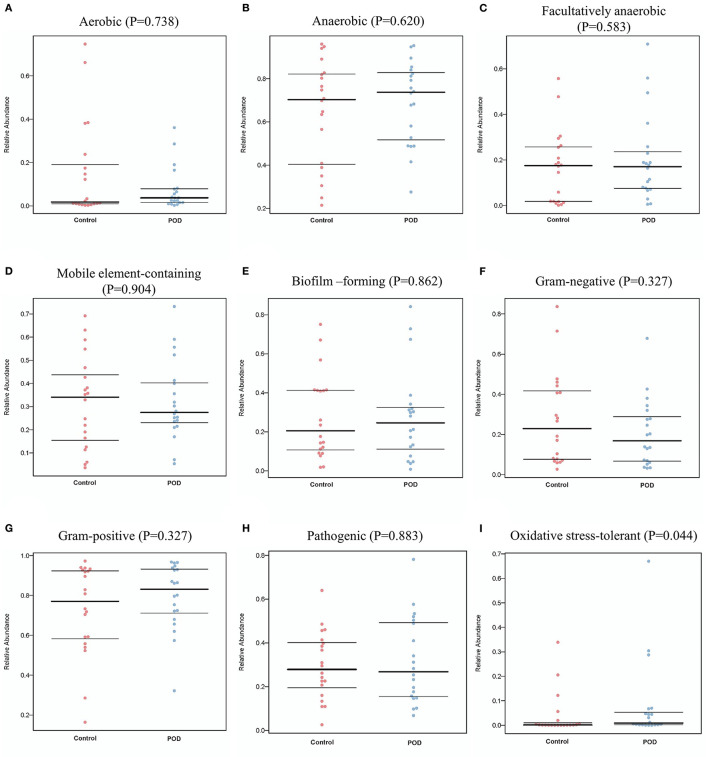
Bacterial phenotype prediction for **(A)** Aerobic, **(B)** Anaerobic, **(C)** Facultatively anaerobic, **(D)** Mobile element-containing, **(E)** Biofilm-forming, **(F)** Gram-negative, **(G)** Gram-positive, **(H)** Pathogenic, and **(I)** Oxidative stress-tolerant.

We also conducted bacterial gene functional prediction through Gene Ontology (GO) and pathway prediction analyses, as illustrated in Figures 4A,B. We predicted the potential phenotypes of the gut microbiota, including aerobic, oxygen-utilizing (anaerobic and facultatively anaerobic), mobile element-containing, biofilm-forming, gram-negative, gram-positive, pathogenic, and oxidative stress-tolerant bacteria (Figures 5A–I). We found a positive association between the phenotype of oxidative stress-tolerant and POD with borderline significance (*P* = 0.044).

## Discussion

We explored the association between the composition of the gut microbiota and POD. Interestingly, we found differential abundances of the gut microbiota between the POD and control cohorts. *Proteobacteria, Enterbacteriaceae, E. shigella, Klebsiella, Ruminococcus, Roseburia, Blautia, Holdemanella, Anaerostipes, Burkholderiaceae, Peptococcus, Lactobacillus*, and *Dorea* were more abundant in the POD cohort, whereas *S. equinus* and *B. hominis* were more abundant in the control cohort.

In recent decades, the “brain-gut” axis has received considerable attentions in clinical association and mechanism studies of neuropsychiatric disorders. Dysregulation of the gut microbiota was reportedly associated with various neuropsychiatric diseases, such as Alzheimer's disease (28), Parkinson's disease (29), ASD (30), and depression (31). Previous studies suggested that the gut microbiota influences the physiological functions of the brain through several pathways. For example, dysregulation of the gut microbiota leads to abnormal activation of systemic and neuro-inflammation (32, 33). It has been reported that 70–80% of immunocytes reside at the gut-associated lymphoid tissue (34), and the gut microbiota is closely related to the inflammatory status. Surgery may induce alterations in the composition of gut bacteria which subsequently secrete endotoxin, leading to endotoxemia and excessive intestinal inflammation, as well as aggravating systemic and neuro-inflammation (35, 36). Kawano et al. (37) have been found that the neuro-inflammation was significantly correlated with delirium. Seo et al. (38) also suggested that neutrophil-lymphocyte ratio could be deserved as a biomarker in delirium. We speculated that the alteration of gut bacteria related neuro-inflammation after abdominal surgery would be a potential influencing factor in the incidence of POD. Additionally, deregulation of the gut microbiota regulates brain function by altering bacterial metabolites. Some neurotransmitters, such as γ-aminobutyric acid, serotonin and dopamine, are produced in the gastrointestinal tract by gut bacteria (39) and reach the central nervous system bypassing the blood-brain-barrier (40, 41). Furthermore, studies have found that anticholinergic medications and dopaminergic medications instigated delirium, indicating that neurotransmitters might play important roles in POD (42, 43). Dysregulation of the gut microbiota in mice using antibiotics led to neural pathological changes, with some neurological diseases more severe than in the non-dysregulation mice cohort (34). Thus, the gut microbiota is important in the pathogenesis of neuropsychiatric disorders. However, few studies have focused on POD.

In a clinical study of the relationship between the gut microbiota and pseudopsia after cardiac surgery, Maekawa et al. (22) reported that surgery lowered the total bacterial counts and species numbers. *Staphylococcus* and *Pseudomonas* counts were significantly higher postoperatively, and may be associated with the pathogenesis of pseudopsia. However, we did not conduct a longitudinal investigation to explore whether differences in the abundance of bacterial species occurred pre- and post-operatively. Numerous studies have suggested that the composition of the gut microbiota changes dramatically after abdominal surgery (44). Localized inflammation and antibiotics administration during abdominal surgery influences the gut microbiota composition. As different surgical methods and diverse antibiotics may confound the postoperative results, we only analyzed the association of the pre-operative gut microbiota status with the onset of POD. Zhang et al. (21) conducted a preliminary study of the association of delirium-like behavior with gut microbiota in an abdominal surgical mouse model. They found that multiple bacterial types were significantly associated with delirium, such as *Gammaproteobacteria, Bifidobacteriales, Ruminococcaceae, Butyricimonas, E. shigella*, and others. These results are partly consistent with our results and indicate that gut microbiota-induced psychological alterations are derived from a common bacterial cohort both in humans and mice. *Gammaproteobacteria* and *E. shigella* are both pathogenic and can colonize the gastrointestinal tract, leading to abnormal activation of gut inflammation. In reviewing previous studies, we did not find the clinical association between of post-operative diarrhea which could be induced by *E. shigella* with the incidence of POD. However, a previous study suggested that Shige toxin from *Shigella* contributed to the pathogenesis of delirium (45). Although *E. shigella* in our study might be a kind of colonized bacterial species and would not cause diarrhea, we speculated that the Shige toxin derived from the colonized *E. shigella* plays a role in the pathogenesis of POD after abdominal surgeries. This might provide clues in the prevention or treatment of POD by the intervention of gut bacteria, such as *E. shigella*. Animal experiments have shown that surgery significantly increased levels of *Gammaproteobacteria* (21), the correlation between the differential abundance of *Gammaproteobacteria* before surgery and POD has not been reported in clinical studies. Some studies suggested that higher proportions of *Gammaproteobacteria* were significantly associated with major depressive disorder (46) and ASD (47). Therefore, there may also be a connection between *Gammaproteobacteria* and POD. Although *Gammaproteobacteria* was found associated with POD in our study, the mechanisms need further analysis. Furthermore, *Ruminococcaceae* was previously reported to be correlated with the anti-depressant effects of R-ketamine in mice (48), however, some *Ruminococcaceae* at the genus level showed higher abundance in POD than in control. These results are different from previous studies of *Ruminococcaceae* in psychological disorders. As we could not find other related references to POD and *Ruminococcaceae*, a more comprehensive investigation of a larger cohort is required to confirm our results regarding the association between *Ruminococcaceae* and POD. We have also developed a model to predict the diagnostic efficacy of bacterial types in POD. Considering the results of differential abundance analysis, that *E. shigella* may predict POD based on its area under the curve of 0.75.

Finally, we performed phenotype prediction based on the differentially abundant bacterial types. The results indicated that oxidative stress tolerance was involved in the pathogenesis of POD. Oxidative stress has been widely reported to be involved in the pathogenesis of POD (49, 50). A detailed mechanism study is needed to investigate whether the production of oxidative stress-related metabolites is correlated with the gut microbiota.

Our study had some limitations. First, we only evaluated the association of POD with the gut microbiota. The detailed mechanisms through which the gut microbiota leads to the pathogenesis of POD were not examined. Secondly, the sample size in our study was relatively small, which may led to bias in statistical analysis. Therefore, additional samples should be evaluated in order to confirm our results in delirium clinical prediction.

## Conclusion

In summary, we found significant associations between the pathogenesis of POD and composition of the gut microbiota. *E. shigella* is a promising diagnostic bacterial species for predicting POD onset after abdominal surgery. Phenotype prediction revealed that the gut microbiota may influence POD through oxidative stress tolerance.

## Data Availability Statement

The datasets presented in this study can be found in online repositories. The names of the repository/repositories, accession number(s) can be found at: NCBI BioProject, PRJNA797529.

## Ethics Statement

The studies involving human participants were reviewed and approved by Ethical Committee of Anhui Medical University, Hefei, Anhui, China. The patients/participants provided their written informed consent to participate in this study.

## Author Contributions

HL, C-hW, and X-sL contributed to conception and design of the study. GC and Y-lX organized the database. QF performed the statistical analysis. HL wrote the first draft of the manuscript. LY wrote sections of the manuscript. All authors contributed to manuscript revision, read, and approved the submitted version.

## Funding

This work was supported by the National Natural Science Foundation of China, Grant Nos. 81870841 and 82101268.

## Conflict of Interest

The authors declare that the research was conducted in the absence of any commercial or financial relationships that could be construed as a potential conflict of interest.

## Publisher's Note

All claims expressed in this article are solely those of the authors and do not necessarily represent those of their affiliated organizations, or those of the publisher, the editors and the reviewers. Any product that may be evaluated in this article, or claim that may be made by its manufacturer, is not guaranteed or endorsed by the publisher.
