# Supplementary material for: Preoperative Status of Gut Microbiota Predicts Postoperative Delirium in Patients With Gastric Cancer
Source: Front Psychiatry. 2022 Mar 3;13:852269. doi: 10.3389/fpsyt.2022.852269 (PMC8929925; doi:10.3389/fpsyt.2022.852269)

Supplementary Figure 1. α-diversity analyses of data. (A) Observed operational taxonomic unit analysis; (B) Good coverage analysis; (C) Chaos analysis; (D) Shannon analysis; (E) Simpson analysis.

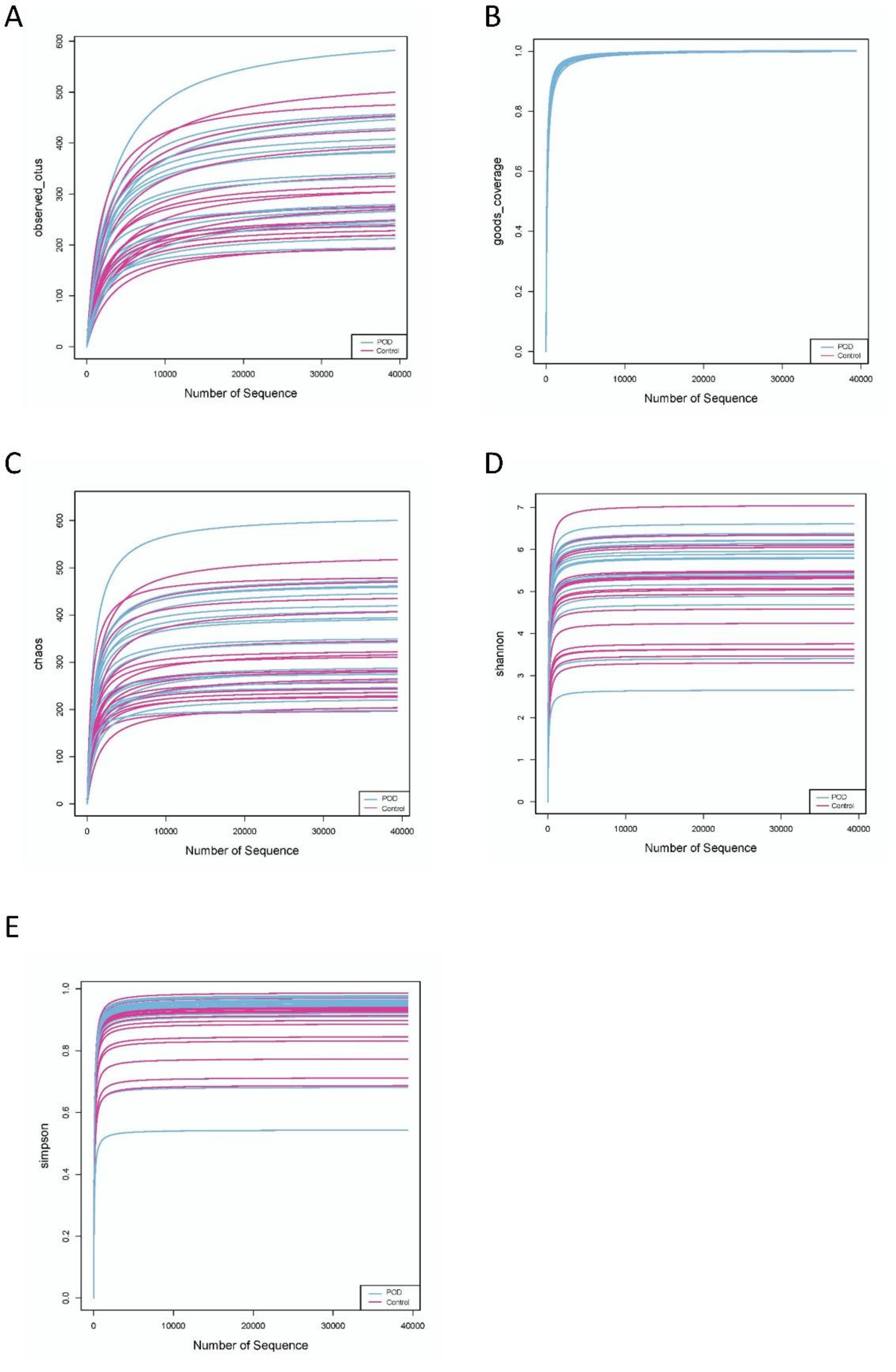

Supplement: Supplementary file 1 [file Presentation_1.pdf]
